# Renin-Angiotensin-Aldosterone System Blockers Prior to Hospitalization and Their Association With Clinical Outcomes in Coronavirus Disease 2019 (COVID-19)

**DOI:** 10.7759/cureus.13429

**Published:** 2021-02-18

**Authors:** Ranjit Banwait, Devina Singh, Anamarys Blanco, Vaibhav Rastogi, Khalid Abusaada

**Affiliations:** 1 Internal Medicine, University of Central Florida College of Medicine/Hospital Corporation of America (HCA) Healthcare, Gainesville, USA; 2 Internal Medicine, University of Central Florida College of Medicine/Hospital Corporation of America (HCA) Healthcare, Ocala, USA; 3 Internal Medicine, Envision Physician Services, Lake City, USA

**Keywords:** raas, ace inhibitors, arbs, covid-19

## Abstract

Objective: To determine the effect of angiotensin-converting enzyme inhibitors (ACEIs) and angiotensin receptor blockers (ARBs) use prior to hospitalization on clinical outcomes in coronavirus disease 2019 (COVID-19) patients.

Design: An observational retrospective cohort study from 178 hospitals from a large health system across the United States.

Patient population: Hospitalized patients (n=2726) with confirmed COVID-19 between January 1, 2020, and April 1, 2020.

Main outcome(s) and measure(s): Outcomes during hospitalization, including disease severity by level of care, intensive care unit (ICU) admission, mechanical ventilator (MV) use, hospital length of stay, and in-hospital death. Patient demographics and comorbidities were also recorded.

Results: A total of 2,726 patients were included in the analysis. Three hundred ninety-eight (14.6%) patients were taking an ACEI, while 352 (12.9%) patients were taking an ARB prior to hospitalization. After adjusting for comorbidities, age, renal function, and severity of illness based on level of care, ACEI prior to admission was independently associated with decreased need for MV (odds ratio [OR] 0.56, p value 0.003) and mortality (OR 0.45, p value <0.001). Similarly, patients who took ARBs were less likely to require MV when compared to the non-renin-angiotensin-aldosterone system blockade (RAASb) group (7.4% vs 12.2%, p value 0.009, respectively). ARB prior to admission was also independently associated with decreased need for MV (OR 0.46, p value 0.001) and mortality (OR 0.66, p value 0.017) compared to the non-RAASb group.

Conclusion: Taking ACEIs and ARBs prior to admission for COVID-19 was independently associated with decreased need for mechanical ventilation and in-hospital mortality.

## Introduction

The coronavirus disease 2019 (COVID-19) pandemic is caused by the severe acute respiratory syndrome coronavirus 2 (SARS-CoV-2). As of late October 2020, COVID-19 has affected approximately 108 million individuals worldwide, causing an estimated 2.3 million deaths. In the United States alone, more than 27.8 million cases have been reported and the numbers continue to rise at an alarming rate [1]. SARS-CoV-2 is a positive-strand ribonucleic acid (RNA) virus that is believed to enter human cells through binding its viral spike glycoprotein N-terminal portion (S1 domain) to the host angiotensin-converting enzyme 2 (ACE2) receptor, which has a high expression in heart and lung tissues and is part of the renin-angiotensin-aldosterone system (RAAS) [2,3].

The use of RAAS blockers (RAASb) has been controversial after a correspondence by Fang et al. that hypothesized that patients taking inhibitors of the RAAS were more susceptible to COVID-19 infection and had a higher risk of developing severe and fatal complications. They proposed possible upregulation of ACE2 receptors with the use of RAASb and, therefore, increased binding sites for SARS-CoV-2 entrance to the host cell [4]. Conversely, Sun et al. hypothesized that inhibitors of the RAAS produce a disruption of the entire system that could potentially lead to a decrease in the production of ACE2 receptors, reducing the entry of the virus into the cell [5]. Furthermore, the expression of ACE2, an enzyme that has been linked to protective anti-inflammatory properties, is thought to be downregulated by SARS-coronavirus infections, leading to increased activation of RAAS which aids in exacerbating the lung injury; consequently, the use of RAASb could decrease these effects [6].

A recent meta-analysis by Usman et al. showed no association between RAASb use and mortality in COVID-19 patients. However, there was lack of data regarding the separate use of angiotensin-converting enzyme inhibitors (ACEIs) and angiotensin receptor blockers (ARBs) and adjusted data was reported by only one study [7].

The aim of this study is to further the understanding of the role of ACEIs and ARBs in COVID-19 infections and fill in some of the gaps in our knowledge regarding the effect of RAASb on clinical outcomes of COVID-19.

## Materials and methods

Study design

This is a retrospective observational cohort study that utilized the Hospital Corporation of America (HCA) data. HCA is a large heath care system that involves 178 hospitals across the United States. This study was conducted in accordance with the Declaration of Helsinki and approved by the HCA Healthcare Institutional Review Board (IRB) Manager (Protocol no: 2020-173; Dataclear Project no: 2020-1369). The requirement for written informed consent was waived as the obtained data was de-identified.

Data collection and review

All patients hospitalized with COVID-19 (ICD10 U07.1) at one of the HCA hospitals nationwide between January 1, 2020, and April 1, 2020, were included in this study. SARS-CoV-2 was confirmed with polymerase chain reaction (PCR) testing of a nasopharyngeal or oropharyngeal swab. Data were extracted from the enterprise electronic medical records (EMR) by a research analyst who created a de-identified data set. All study records were kept in a password-protected study folder on a closed, enterprise-owned network.

Data elements and outcomes

Data elements included patient demographical information, comorbidities, home medications, vitals and laboratory tests on admission, smoking status, inpatient diagnoses, inpatient medications, treatments, procedures including invasive mechanical ventilation, length of hospital stay (LOS), and mortality.

Home medications, including ACEIs or ARBs, were evaluated based on the admission medication reconciliation by the inpatient-accepting physician. Comorbid conditions included: pulmonary diseases (asthma, chronic obstructive pulmonary disease, pulmonary fibrosis, and obstructive sleep apnea), cardiac diseases (hypertension, hyperlipidemia, coronary artery disease, and congestive heart failure), renal diseases (chronic kidney disease and end-stage renal disease), metabolic disorders (type I and II diabetes mellitus and obesity), liver diseases (chronic liver disease and cirrhosis), autoimmune disorders (osteoporosis, rheumatoid arthritis, lupus, and autoimmune thyroid disease), human immunodeficiency virus positivity, cerebrovascular disease, and cancers.

The primary outcome was mortality, which included all-cause death or hospice discharge. Secondary outcomes included 1) severity of COVID-19, with mild/moderate disease defined as highest level of care being medical floor, critical disease defined as highest level of care being intensive care unit (ICU) and requiring mechanical ventilation and/or vasopressor support, and severe disease defined as highest level of care being ICU but not meeting criteria for critical disease; and 2) length of hospital stay. These outcomes were recorded for patients who completed their hospital course at the end of the study period (April 1, 2020). 

Statistical analysis

Patients were divided into three groups. Patients who took ACEIs prior to hospitalization (ACEIs group), patients who took ARBs prior to hospitalization (ARBs group), and patients who took neither ACEIs nor ARBs prior to hospitalization (non-RAASb group). Characteristics and outcomes of ACEIs and ARBs vs non-RAASb patients were compared in bivariate analysis. Outcomes were further analyzed via linear and logistic regression models using demographic characteristics, number of comorbidities, creatinine level, and severity of illness to further characterize the independent effect of ACEIs and ARBs on outcomes. All data analysis for the characteristics and outcomes was presented as a mean and standard deviation for continuous data or as a percentage for categorical data. All statistical analysis with a P-value of <0.05 was considered statistically significant. The statistical program used for analysis was STATA, version 15.0 (StataCorp, College Station, TX, USA).

## Results

**Figure 1:**
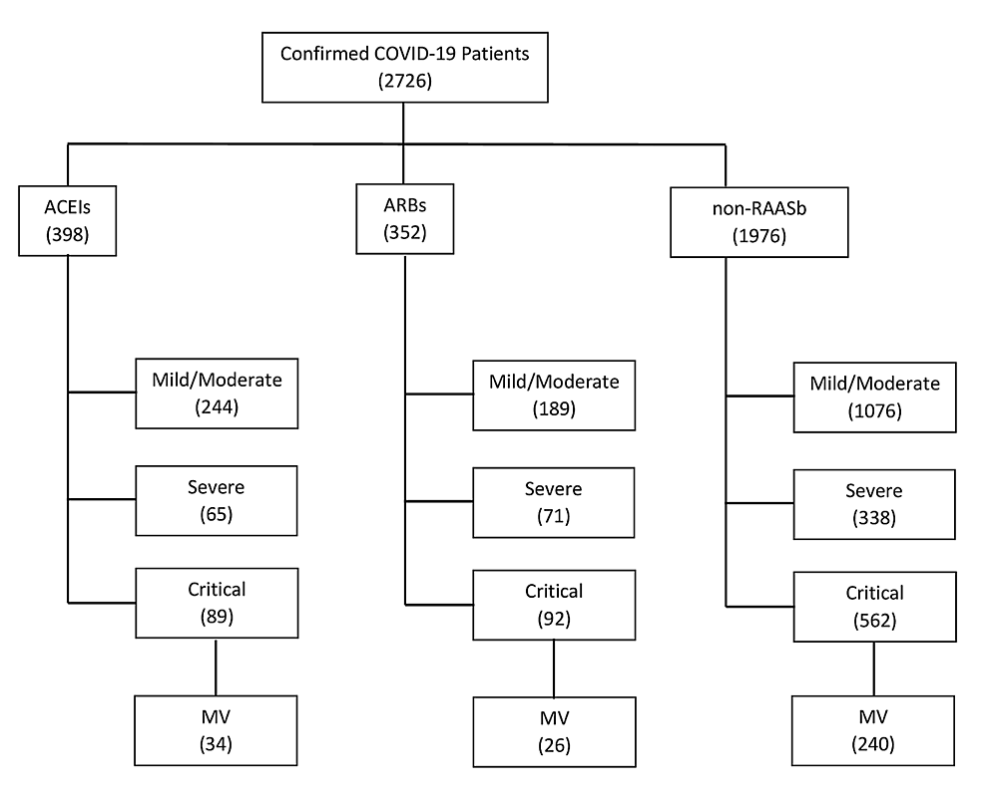
Flow diagram of the study population Angiotensin-converting enzyme inhibitors (ACEIs), Angiotensin receptor blockers (ARBs), Coronavirus disease 2019 (COVID-19), non-renin-angiotensin-aldosterone system blockers (RAASb), Mechanical ventilation (MV)

In the present study, 2,726 consecutively confirmed COVID-19-positive patients were included. Patients were stratified into ACEIs, ARBs, and non-RAASb groups (Figure 1). Of those, 398 (14.6%) patients were taking ACEIs and 352 (12.9%) were taking ARBs. When compared with the non-RAASb group, patients on ACEIs and ARBs were significantly older and had more comorbidities compared with the non-RAASb group (Table 1).

**Table 1:** Demographical information of study population Angiotensin-converting enzyme inhibitors (ACEIs), Angiotensin receptor blockers (ARBs), Coronavirus disease 2019 (COVID-19), non-renin-angiotensin-aldosterone system blockers (RAASb), Intensive Care Unit (ICU), Blood urea nitrogen (BUN). Chi-square test is used to calculate the p-values.

	Total (n=2726)	Non-RAASb (n=1976)	ACEIs (n=398)		ARBs (n=352)	
			N, %	P value (ACE vs non-RAASb)	N, %	P value (ARB vs non-RAASb)
Age (mean years)	60.65	58.37	65.59	<0.001	67.55	<0.001
Sex				0.209		0.003
Female	1274 (46.74)	912 (46.15)	170 (42.71)		192 (54.55)	
Male	1452 (53.26)	1064 (53.85)	228 (57.29)		160 (45.45)	
Race				0.051		0.029
Caucasian	1303 (47.80)	926 (46.86)	192 (48.24)		185 (52.56)	
African American	689 (25.28)	485 (24.54)	114 (28.64)		90 (25.57)	
Other	734 (26.93)	565 (28.59)	92 (23.12)		77 (21.88)	
Smoking status				0.014		0.013
Never smoker	1765 (64.75)	1293 (65.44)	240 (60.30)		232 (65.91)	
Ever smoker	633 (23.22)	428 (21.66)	113 (28.39)		92 (26.14)	
Unknown	328 (12.03)	255 (12.90)	45 (11.31)		28 (7.95)	
Number of comorbidities (mean num)	2.05	1.75	2.81	<0.001	2.85	<0.001
Laboratory markers (mean mg/dL)						
BUN	22.31	21.92	22.90	0.447	23.84	0.165
Serum creatinine	1.22	1.22	1.18	0.688	1.23	0.875
COVID-19 disease severity				0.017		0.310
Mild/Moderate	1509 (55.36)	1076 (54.45)	244 (61.31)		189 (53.69)	
Severe	474 (17.39)	338 (17.11)	65 (16.33)		71 (20.17)	
Critical	743 (27.26)	562 (28.44)	89 (22.36)		92 (26.14)	
ICU level of care	1217 (44.64)	900 (45.54)	154 (38.69)		163 (46.30)	
Mechanical Ventilator use				0.040		0.009
No	2426 (88.99)	1736 (87.85)	364 (91.46)		326 (92.61)	
Yes	300 (11.01)	240 (12.15)	34 (8.54)		26 (7.39)	
Length of hospital stay (mean days)	9.53	9.33	9.86	0.212	10.27	0.033
Died in hospital	608 (22.30)	456 (23.08)	65 (16.33)	0.003	87 (24.72)	0.512

Primary outcome

Overall, 608 (22.3%) patients either died or were discharged to hospice care. Mortality in the ACEIs group was significantly lower when compared to the non-RAASb group (n=65, 16.3%; p value 0.003) (Table 1). When adjusted for severity of illness, age, race, number of comorbidities, and creatinine level, the ACEIs group was independently associated with decreased mortality (odds ratio [OR] 0.454, 95% CI 0.318-0.647, p value <0.001).

In the ARBs group, there was no difference in the unadjusted mortality when compared to the non-RAASb group (24.7% vs 23%, p value 0.512). However, when adjusted for confounders, ARBs use prior to hospitalization was independently associated with decreased mortality (OR 0.660, 95% CI 0.469-0.927, p value 0.017). Other variables that were associated with increase in mortality were age, number of comorbidities, serum creatinine and disease severity (Table 2).

**Table 2:** Multivariate analyses of factors associated with mechanical ventilator use, hospital length of stay, and mortality in COVID-19 patients (N=2,726) Angiotensin-converting enzyme inhibitors (ACEIs), Angiotensin receptor blockers (ARBs), Coronavirus disease 2019 (COVID-19), non-renin-angiotensin-aldosterone system blockers (RAASb), African American (AA)

	Mechanical Ventilator use			Hospital Length of Stay			Mortality		
	OR	95% CI	P value	βcoefficient	95% CI	P value	OR	95% CI	P value
Age	1.004	0.996 – 1.012	0.324	0.052	0.036 – 0.69	<0.001	1.083	1.072 – 1.093	<0.001
Race Caucasian vs AA	1.008	0.848 – 1.198	0.923	-0.279	-0.640 – 0.080	0.128	1.005	0.844 – 1.197	0.950
Smoking status Ever vs Never smoker	0.972	0.694 – 1.241	0.615	0.420	-0.193 – 1.034	0.180	0.981	0.747 – 1.287	0.892
Number of Comorbidities	1.187	1.091 – 1.292	<0.001	0.228	0.359 – 0.421	0.020	1.171	1.079 – 1.270	<0.001
Serum Creatinine	0.972	0.900 – 1.050	0.482	-0.270	-0.430 - -0.111	0.001	1.154	1.084 – 1.230	<0.001
Disease Severity									
Severe vs Mild/Moderate	NA	NA	NA	2.676	1.973 – 3.379	<0.001	1.776	1.244 – 2.536	0.002
Critical vs Mild/Moderate	NA	NA	NA	7.851	7.243 – 8.459	<0.001	20.263	15.228 – 26.962	<0.001
ACEIs vs Non-RAASb	0.555	0.377 – 0.816	0.003	0.343	-0.414 – 1.102	0.374	0.454	0.318 – 0.647	<0.001
ARBs vs Non- RAASb	0.465	0.302 – 0.716	0.001	0.270	-0.526 – 1.066	0.506	0.660	0.469 – 0.927	0.017

Secondary outcomes

Of the patients taking ACEIs, 244 (61.3%) patients required non-ICU level of care (mild/moderate), while 154 (38.7%) patients required ICU (severe/critical). Similarly, in the ARBs group, 189 (53.7%) patients required non-ICU level of care (mild/moderate), and 163 (46.3%) patients required ICU (severe/critical). When compared to patients in the non-RAASb group, the ACEIs group had less patients in the increased severity groups (critical severity: ACEIs=22.36% vs non-RAASb=28.47%, p value 0.017). Although a lower percentage of patients were noted to be in critical severity in the ARBs group when compared to the non-RAASb group (ARBs=26.14% vs non-RAASb=28.47%), no statistical significance was found (p value 0.310) (Table 1). 

When compared to the non-RAASb group, both the ACEIs and ARBs groups had decreased use of mechanical ventilation (ACEIs=8.5%; ARBs=7.4%; non-RAASb=12.2%) (Table 1). When adjusted for confounders, both ACEIs (OR 0.555, 95% CI 0.377-0.816, p value 0.003) and ARBs (OR 0.465, 95% CI 0.302-0.716, p value 0.001) were independently associated with decreased need for mechanical ventilation (Table 2). 

Mean length of stay (LOS) in hospital was 9.5 days. There was no difference in LOS between the ACEIs and non-RAASb groups (ACEIs=9.86; non-RAASb=9.33, p value 0.212). The ARBs group was noted to have a longer LOS when compared to the non-RAASb group (ARBs=10.27, non-RAASb=9.33, p value 0.033) (Table 1). However, when adjusted for confounders, no statistical difference was found in hospital length of stay for the ACEIs and ARBs groups when compared to the non-RAASb group (ACEIs p value 0.374; ARBs p value 0.506) (Table 2).

## Discussion

In this observational retrospective study comprising 2,726 confirmed COVID-19-positive patients, we found that use of ACEIs and ARBs prior to hospitalization was associated with decreased mortality, decreased severity of illness, and need for mechanical ventilation.

As expected, patients on these medications were significantly older and had a substantially higher number of total comorbidities. Despite this, there was a significant difference in the mortality rate in patients taking ACEIs (OR 0.37, p value <0.001) and ARBs (OR 0.50, p value 0.017) when compared to patients on neither of these medications prior to hospitalization.

Previous studies evaluating the impact of RAASb use prior to hospitalization on COVID-19 outcomes have been mixed. A study on a Chinese population by Zhou et al. evaluated 3,572 patients with ACEIs/ARBs in COVID-19 and found that ACEIs/ARBs were associated with improved 28-day in-hospital mortality with no significant difference between the ACEIs and ARBs groups [8]. Meng et al. reported a lower severity of COVID-19 cases, decreased interleukin-6 levels and peak viral load along with increased CD3 and CD8 T cell counts in their COVID-19-positive patient population of 42 [9]. On the other hand, in a large case-control study from Italy, Mancia et al. found no association between use of ACEIs or ARBs with severe and critical presentation of COVID-19 [10]. Similarly, a study by Richardson et al. reported outcomes in the United States and found no difference in mortality between ACEIs/ARBs and non-RAASb groups [11]. However, in both of these studies the data was not adjusted for confounders, like age and comorbidities, which may have been responsible for the findings. Furthermore, Mehta et al. conducted a study in which they reported that ACEIs were associated with higher probability of worse clinical outcomes. However, again the analysis was unadjusted for confounders [12].

A recent meta-analysis by Usman et al. encompassing eight studies (n=62,706), including studies mentioned above, showed no association between RAASb use and mortality in COVID-19 patients [7]. Another meta-analysis by Grover et al. also showed no association of ACEIs/ARBs with disease severity and mortality compared to non-users [13]. In both of these analyses, the authors advised to view the results with caution as there was lack of data regarding use of ACEIs and ARBs separately and adjusted data was reported by only one study. Patients on ACEIs and ARBs in these studies were notably older and had higher burden of comorbidities, which may have confounded the results of the meta-analyses.

ACEIs and ARBs effects on ACE2 expression, especially in the lungs, are still not very clear [14]. A small study involving 12 COVID-19 patients looked at biochemical markers in COVID-19 infection and observed significant elevation in angiotensin II levels in the plasma, which was linearly related to total viral load and lung injury [15]. Although the sample size of this study was too small to make any meaningful association, this could be a plausible explanation for ARBs' beneficial effect in COVID-19 patients seen in our study, as it inhibits the angiotensin II receptor type 1 which is responsible for the deleterious effects of angiotensin II. In fact, there are two clinical trials underway evaluating the efficacy of losartan in hospitalized (NCT04312009) and non-hospitalized patients (NCT04311177).

Our findings support the recommendations from the World Health Organization, the American College of Cardiology/American Heart Association/Heart Failure Society of America, and the European Society of Cardiology declaring to continue the use of ACEIs/ARBs as prescribed for the guidelines as there is no evidence of increased severity of COVID-19 illness [16,17]. It also provides assurance to physicians to prescribe ACEIs/ARBs if indicated to their patients without fear that it would worsen outcomes should they get COVID-19. Furthermore, based on our study, their use could be beneficial by decreasing severity of illness and mortality, although this needs to be confirmed by randomized controlled trials.

Strength and limitations

The main strength of our study is its generalizability. Data is inclusive of 178 hospitals across the USA with a COVID-19-positive patient population equaling 2,726. The study comprised almost 50% Caucasian patients with the remaining being African Americans and other races. The numbers of females and males in our study are comparable. The outcomes in our study are adjusted for comorbidities, age, race, and renal function.

Study limitations include, first, that despite this large COVID-19-positive patient population, we were only able to identify 398 patients taking ACEIs and 352 patients taking ARBs in the outpatient setting prior to being hospitalized. Second, this is an observational retrospective cohort study, thus any associations found can’t be taken as causal relationship between ACEIs/ARBs and mortality or severity of disease. Third, oropharyngeal and nasopharyngeal swabs were used in COVID-19 detection and both tests have different sensitivity and specificity, which might result in variation in number of false positives or negatives. Fourth, although all potential confounders were accounted for, difference in intervention during the hospitalization may have accounted for some of the effects seen. Fifth, medication lists were obtained from the electronic medical records. It can’t be ascertained if patients were compliant with the medications and how long patients were on the medications.

## Conclusions

In summary, ACEIs and ARBs are independently associated with decreased mortality and mechanical ventilation in hospitalized patients with COVID-19. Large scale randomized controlled trials are essential to evaluate the causal relationship between ACEIs/ARBs and morbidity and mortality of COVID-19. The conclusion of this study is in line with the recommendations of professional organizations throughout the world.
